# Annotations

**Published:** 1886-11-13

**Authors:** 


### Annotations.

**Hospitals: Provident Dispensaries and the Pay System.**

The advocates and opponents of payments by hospital patients have for some time enjoyed a truce in the metropolis. Each party is taking time to digest the arguments of the other, and each, it may be, is thinking of buckling on its armour afresh for attack or defence. For the time, the struggle is transferred to the provinces, and Liverpool is resounding with the din of battle. In that city there is a strong feeling among some that a system of small payments by out-patients, or the affiliation of provident dispensaries to the out-patient department of the several hospitals, or the complete conversion of that department into a system of *provident* rather than *free* relief, is desirable. There is evidently a desire for some bold and sweeping change, which shall effectually relieve the burdened finances of the hospitals. Mr. Robert Rentoul is the energetic advocate of change, and his letters on the subject, which have recently appeared in several Liverpool papers, are worthy of careful consideration. Mr. Rentoul gives particulars of different modifications of the pay system, as practised in London, Manchester, Devonport, and other places. In Manchester there is active co-operation between the hospitals and certain provident dispensaries, with the object of drafting off from the hospitals to the dispensaries all those out-patients who, by reason of their sufficient wages, are not deemed eligible for charitable relief. There is a wage-limit, and a body of inspectors, acting in the interest of, and paid by the provident dispensaries. According to this system every applicant for out-patient relief is treated at least once. In the interval he is visited by an inspector, and if he is found to be above the wage-limit, his ticket is stamped, he is informed that he cannot again be treated at the hospital, and is advised to attend at one of the provident dispensaries, to which his stamped hospital-ticket is an immediate passport. If, however, he persists in returning to the hospital, the stamped ticket shows him to be above the wage-limit, and he is refused further treatment. In this scheme there are several important points. In the first place the hospital neither demands nor receives payment from its out-patients. In the next place every applicant is treated once before inquiry is made, and thus no one has to wait, and no really needy person is made to suffer. Thirdly, the wage-limit being a fixed point, nothing is left to the discretion of the inspector, and so there can be no favouritism and no hardship. The Manchester scheme has been found to work exceedingly well. The congested out-patient department has been relieved, the burden upon the hospital finances greatly diminished, and the prosperity of the provident dispensaries as greatly promoted. Mr. Saunders, secretary to the Infirmary, Mr. Macdonald, clerk to the Guardians, and Mr. Forrest, hon. secretary to the Ancoats Hospital, agree in speaking of it as a "manifest saving to the charities." It has also tended to promote self-respect among the poor, for whilst in 1875 the percentage of ineligible applicants at the out-patient department was 43.32, in 1885 it had fallen to 13.57, a most remarkable and gratifying diminution. The whole question of out-patient relief is of the first importance, and will be returned to from time to time as opportunity offers.

**Hospital Sunday in Birmingham.**

Hospital Sunday was founded in Birmingham, and on the 1st inst., for the twenty-eighth time, the congregations worshipping in Birmingham and the neighbourhood were appealed to simultaneously for contributions on behalf of the hospitals. The Hospital Sunday collections in Birmingham are devoted one year to the General Hospital, the next to the Queen's Hospital, and the third year the sum contributed is divided amongst the other medical and surgical charities of the town. It was the pressing pecuniary necessities of the Birmingham General Hospital which led the late Canon Miller and Mr. Thomas Wright to organise simultaneous collections on the first occasion in aid of that charity. For all these reasons, it is interesting to ascertain how far the spirit of the founders towards the hospitals continues to animate the inhabitants of Birmingham at the present time. It will be seen from the statement in our Notes and News column, that in 1859 the sum raised was £5,200, although the population at that date was probably at least 100,000 less than it is to-day. Last year (1885) the amount collected was only £4,773, and the returns received so far lead us to fear that this year the total contributions will be less than last. The largest sum ever raised in Birmingham was in 1878, when £6,482 was paid over to the treasurer of the Queen's Hospital. In these circumstances, if depression of trade has had anything to do with this great falling off in the contributions to the Birmingham Hospital Sunday Fund, the managers of the movement in London have solid ground for satisfaction in the fact that, under much more adverse circumstances, the inhabitants of the metropolis contributed this year several thousand pounds more than on any previous occasion. How is it that Birmingham has so sadly fallen away from the high position and foremost place which it used to occupy as the birthplace of Hospital Sunday?

**Hospital Sunday Practices to be avoided.**

A correspondent of the *Birmingham Gazette* states that a practice has arisen of late years in connection with the Birmingham Hospital Sunday collections which is altogether a departure from the first intention of its founders. This practice consists in making special donations to particular institutions, not included in the year's collection, which have participated already in their proper turn in the triennial period. He points out that anyone can give what and when he pleases direct to the object that he most favours, and can get his gifts publicly acknowledged in the newspapers within a few days. He ascribes the reason for this harmful departure from a useful rule to a desire to glorify a particular place of worship. Another abuse pointed out is the publication of the name of every donor who gives one guinea or more —a practice which he declares to be full of evil and rancour, because it sets some members of each congregation in a different class from the remainder. As the Duke of Cambridge says elsewhere, it is not the amount of the gift, but the sentiment which inspires it, which is the important thing to keep in mind. Whether there has been any departure from this high standard in Birmingham or not, leading to the serious falling off in the contributions of the inhabitants on Hospital Sunday, we cannot, of course, say; but whatever the cause may be, we hope the local press will take the matter up, as the metropolitan press took up the same question in London this year. If this is done, the result will be, we are certain, that Birmingham will once more prove that her citizens are neither less liberal nor more prone to neglect the medical charities than are the inhabitants of London, Liverpool, or any other great city.

**The Asylums Board and Clinical Teaching.**

The asylums under the control of the Metropolitan Asylums Board have long been recognised as valuable training-grounds for medical students wishing to obtain a knowledge of fevers. Though fevers of various kinds furnish a large portion of the ordinary practice of medical men, the facilities for their practical study are in many cases almost nil. Whilst a student has no difficulty in obtaining a knowledge of such specialties as the treatment of eyes, ears, teeth, etc.—specialties which he may hardly ever require to use—he can scarcely obtain any practical knowledge of fevers, during his hospital curriculum, for love or money. The folly of this is pitiable. On Saturday last, with the sanction of the Local Government Board, the Asylums Board, acting on the advice of Mr. Scovill, decided to open their institutions for the purposes of clinical instruction. So far, well. But why do they stand, like the "damsel named Rhoda," opening their doors such a very little way that no one but fully qualified and registered practitioners can get in? Surely students, and especially senior students, need to have a knowledge of fevers; and seeing that nearly all the fevers in London go to the hospitals under the Board's authority, where are they to get it except at these hospitals? Whilst heartily approving of the action of the Board in thus partially opening their doors, we would venture to say to them, "Throw your doors wide open, and let any young man enter, under necessary restrictions, who gives evidence of a desire and determination to devote himself to real and earnest work." There is no risk to be run in such a course, and the advantages to young doctors and the public would be incalculable.

